# 

**Published:** 1896-12

**Authors:** 


### CONTENTS.

PROCEEDINGS.

Address at the Opening of the Session of 1896-97........................ 573

President's Address........................................................ 582, 592

Points of Interest in the Care of Children's Teeth.................................. 600

COMMUNICATIONS.

The Disinfection of Pulpless Teeth.......................................... 584

Electro-Therapeutics.......................................................... 590

Monthly Digest ................................................................ 609

SELECTIONS.

Customs of Russian Doctors.................................................. 589

Treatment of Pyorrhœa Alveolaris............................................. 591

Hallucination and Anæsthetics................................................ 608

Notes on Tuberculosis—Avenues of Invasion.................................... 614

French Physicians on the Drink Problem......................................... 615

